# *Clostridium difficile* Colitis Leading to Reactive Arthritis: A Rare Complication Associated With a Common Disease

**DOI:** 10.1177/2324709618767689

**Published:** 2018-03-30

**Authors:** Asghar Marwat, Hassan Mehmood, Ali Hussain, Muzammil Khan, Asad Ullah, Medha Joshi

**Affiliations:** 1 Temple University, Philadelphia, PA, USA; 2 Conemaugh Memorial Medical Center, Johnstown, PA, USA

**Keywords:** *Clostridium difficile* infection, reactive arthritis

## Abstract

The relationship between reactive arthritis and enteric infections caused by *Yersinia enterocolitica, Campylobacter jejuni*, and *Salmonella typhimurium* is well documented. *Clostridium difficile* colitis is a less recognized cause of reactive arthritis. We present a case of a 58-year-old woman with *Clostridium difficile* colitis complicated by reactive arthritis. A 58-year-old woman with no significant past medical history presented to our hospital with complaints of nonbloody watery diarrhea, abdominal pain for the past 1 week, and right knee pain starting 1 day prior. The patient had recently used antibiotics for a respiratory tract infection. On examination, the patient had a swollen and erythematous right knee. While in the hospital the patient also developed a similarly painful and swollen left knee. The patient was found to be positive for *C difficile* toxin in stool. Synovial fluid analysis of both the knee joints revealed a sterile and inflammatory fluid, negative for crystals and showing no growth on gram stain. We diagnosed the patient with reactive arthritis secondary to *C difficile* colitis once all other causes of the bilateral knee joint symptoms were ruled out with appropriate laboratory and imaging studies. Treatment with oral vancomycin and an anti-inflammatory was initiated, and the patient had complete resolution of symptoms. This case illustrates the importance of recognizing *C difficile* colitis as a potential differential for reactive arthritis under the appropriate circumstances. The treatment of reactive arthritis is mainly supportive and treating the underlying cause, which happens to be *C difficile* in this case.

## Introduction

Reactive arthritis typically manifests as an acute aseptic, inflammatory, asymmetric oligoarthritis commonly affecting the large joints of the lower extremities. Associated extra-articular findings including conjunctivitis, uveitis, enthesopathy, urethritis, balanitis, and keratoderma blennorrhagicum may also be observed.^1,2^ Reactive arthritis usually develops after an infection in a distant part of the body, and the causative organism is never isolated from the joint.^3^ The relationship between reactive arthritis and enteric infections caused by *Yersinia enterocolitica, Campylobacter jejuni*, and *Salmonella typhimurium* is well documented. *Clostridium difficile* colitis is a less recognized because of reactive arthritis.^4^ Only 50 cases have been reported^5^ since it was first described in 1976 by Rollins and Moeller.^6^ We present a case of a 58-year-old woman with *C difficile* colitis complicated by reactive arthritis.

## Case Report

A 58-year-old woman with no significant past medical history came to our hospital with complaints of abdominal pain, diarrhea, and right knee pain. The patient had an acute onset of severe, nontraumatic right knee pain, with redness and swelling of the joint for the past day. She has nonbloody, watery diarrhea going on for the past 1 week with maximum episodes up to 10 in a day. She recently suffered from an upper respiratory tract infection 3 weeks ago before this presentation and completed 10 days of oral cefuroxime subsequently. On physical examination, she was afebrile with a temperature of 99.5°F, blood pressure of 156/68 mm Hg, and pulse of 87 beats per minute. She had red, hot, and swollen right knee with restricted range of motion during extension. There was tenderness noted in the right lower abdominal quadrant with positive bowel sounds. Rest of the physical examination was unremarkable. Initial laboratory studies demonstrated white blood cell count of 11 700/µL, with 13% bands, 14% atypical lymphocytes, platelets of 237 000/µL, an erythrocyte sedimentation rate of 140 mm/h, and C-reactive protein of 27.5 mg/dL. *C difficile* toxin gene B was positive in stool, and oral vancomycin was started. Rest of the stool studies including testing for ova, parasites, and stool culture were negative. Rheumatoid factor, antinuclear antibody, anticyclic citrullinated peptide studies, serology for Lyme titer, and uric acid level were unremarkable. HLA B-27 came back positive. X-ray of right knee showed joint effusion. Arthrocentesis of the right knee revealed white blood cell count of 6100/µL with 86% neutrophils. Synovial fluid crystal examination, gram stain, and cultures were negative. Her pain was getting worse, and the next day she had mild to moderate discomfort in the left knee with restricted range of motion. Aspiration of the left knee was done, and it was also negative for crystals, gram stain, and cultures. Once synovial fluid was confirmed to be sterile, due to continued debility, both knees were injected with 10 mg dexamethasone resulting in significant relief. The patient was started on ibuprofen 375 mg 3 times a day for 4 weeks and vancomycin was prescribed for 14 days in total. Her diarrhea resolved in 3 days along with significant improvement in pain and ambulation. She was discharged subsequently and seen in the clinic after 4 weeks later with the knee pain resolved and return to full functional capacity.

## Discussion

*Clostridium difficile* carries a significant disease burden in the United States. According to the Center for Diseases Control and Prevention, *C difficile* was responsible for almost half a million infections, resulting in roughly 29 000 deaths in 2011.^7^ *C difficile* infection most commonly leads to pseudomembranous colitis, which presents itself as fever and diarrhea starting from 4 to 9 days after starting antibiotic treatment.^8^ Extracolonic manifestations of *C difficile* including bacteremia, osteomyelitis, visceral abscess, empyema, small bowel disease, and reactive arthritis are less frequent and rarely reported.^8^ Our case adds to the growing body of literature on this subject. The hypothesized pathogenesis of reactive arthritis following an enteric infection with *C difficile* is postulated to be an autoimmune response to bacterial antigens in joints and other tissues that gain access into the bloodstream via the intestinal mucosa.^9^ Our patient fulfilled the criteria for the diagnosis of *C difficile* reactive arthritis as established by Putterman and Rubinow in 1993^9^: a sterile inflammatory arthritis with preceding diarrhea and prior antibiotic exposure^2^; stool test positive for *C difficile* toxin^10^; and no alternative diagnosis for arthritis or diarrhea.^11^ With the increased use of antibiotics in recent times and the associated increased incidence of *C difficile* colitis, many rare manifestations of this disease process that were previously unrecognized are coming to the forefront. It would be prudent for physicians to consider reactive arthritis secondary to *C difficile* colitis as a differential diagnosis in a patient with otherwise unexplained acute inflammatory arthritis in the right clinical setting of recent antibiotic use and diarrhea. Early diagnosis and appropriate treatment can improve patient outcomes and prevent unnecessary diagnostic procedures. Reactive arthritis secondary to *C difficile* colitis is managed conservatively with treatment primarily focused on eradicating the *C difficile* infection and has an excellent long-term prognosis.

## Conclusion

Given the expected rise in the incidence of *C difficile* infection, both internist and rheumatologist should include this pathogen in the differential diagnosis of the enteric organism responsible for reactive arthritis. We suspect *C difficile* reactive arthritis may be underrecognized and recommend raising awareness in the health care profession to test *C difficile* toxin in undifferentiated arthritis patients.
